# Hemorrhagic abdominal pseudocyst following ventriculoperitoneal shunt: a case report

**DOI:** 10.1186/s12893-021-01161-y

**Published:** 2021-03-21

**Authors:** Hong-Cai Wang, Yi-Lei Tong, Shi-Wei Li, Mao-Song Chen, Bo-Ding Wang, Hai Chen

**Affiliations:** 1 Department of Neurosurgery, Li Hui Li Hospital of Medical Centre of Ningbo, No. 1111 Jiangnan Road, Yinzhou District, Ningbo, 315041 China; 2 Department of Internal Medicine, Ningbo Huamei Hospital University of Chinese Academy of Sciences, No. 41 Northwest Street, Ningbo, 315040 China

**Keywords:** Case report, Complication, Hydrocephalus, Pseudocyst, Ventriculoperitoneal shunt

## Abstract

**Background:**

Abdominal cerebrospinal fluid (CSF) pseudocyst is an uncommon but important complication of ventriculoperitoneal (VP) shunts. While individual articles have reported many cases of abdominal CSF pseudocyst following VP shunts, no case of a hemorrhagic abdominal pseudocyst after VP shunts has been reported so far.

**Case presentation:**

This article reports a 68-year-old woman with a 4-month history of progressive abdominal pain and distention. She denied any additional symptoms. A VP shunt was performed 15 years earlier to treat idiopathic normal pressure hydrocephalus and no other abdominal surgery was performed. Physical examination revealed an elastic palpable mass in her right lower abdomen, which was dull to percussion. Abdominal computed tomography (CT) scan indicated a large cystic collection of homogenous iso-density fluid in the right lower abdominal region with clear margins. The distal segment of the peritoneal shunt catheter was located within the cystic mass. Abdominal CSF pseudocyst was highly suspected as a diagnosis. Laparoscopic cyst drainage with removal of the whole cystic mass was performed, 15-cm cyst which found with thick walls and organized chronic hematic content. No responsible vessel for the cyst hemorrhage was identified. No further shunt revision was placed. Histological examination showed that the cyst wall consisted of outer fibrous tissue and inner granulation tissue without epithelial lining, and the cystic content was chronic hematoma. The patient had an uneventful postoperative course and remained asymptomatic for 8-mo follow-up.

**Conclusion:**

To the best of our knowledge, this is the first report of hemorrhagic onset in the abdominal pseudocyst following VP shunt. Such special condition can accelerate the appearance of clinical signs of the abdominal pseudocyst after VP shunts, and its mechanisms may be similar to the evolution of subdural effusion into chronic subdural hematoma (CSDH).

## Background

Placement of a ventriculoperitoneal (VP) shunt is an established procedure for the treatment of hydrocephalus of diverse causes; with increasing longevity following successful treatment, however, more complications arise [1]. Extracranial complications of VP shunt usually include tube disconnection, infection, omental clogging, abdominal visceral perforation, and bowel obstruction. Abdominal cerebrospinal fluid (CSF) pseudocyst is an uncommon but important complication, with its incidence ranging from less than 0.33% to 6.8% [2, 3]. It is characterized by a fluid filled collection of CSF in the peritoneal cavity containing the distal end of VP shunt catheter and is surrounded by a wall composed of fibrous tissue without epithelial lining [3]. Such complication can result in an increased pressure within the fluid collection, reducing forward pressure gradient and optimal shunt function [4]. Abdominal CSF pseudocyst has been well documented, but no case of a hemorrhagic abdominal pseudocyst after VP shunt has been reported so far [2–4]. This article reports the first case of a hemorrhagic abdominal pseudocyst after VP shunt and then explores its possible mechanisms. Sharing our experience and typical clinical data may help improve awareness of this special condition.

## Case presentation

A 68-year-old woman presented to the emergency department with a 4-month history of progressive abdominal pain and distention. She began with mild abdominal discomfort and did not care about it at first. Abdominal pain was then aggravated gradually to moderate level and restricted to the right lower abdominal region. She was awake and denied any additional symptoms such as headaches, nausea, vomiting, decreased appetite, constipation, fever, or chills. In addition, no neurological change was observed. A VP shunt with Strata^®^ (Medtronic) programmable valve system was placed 15 years earlier to treat idiopathic normal pressure hydrocephalus and the valve pressure was 1.5. The patient had no history of abdominal blunt trauma, malignancy, pancreatic or liver diseases. No other abdominal surgery or shunt revision was performed.

Physical examination revealed an elastic palpable mass in her right lower abdomen, which was dull to percussion. Her abdomen was minimally tender in the right lower abdominal quadrant, but no rigid or tense. No skin changes overlying her shunt catheter and no signs of intracranial hypertension were found. A lumbar puncture was performed to analyze CSF. CSF pressure, nucleated cells and protein count were normal; culture of the CSF was also negative for microorganisms. In addition, no evidence of infection was found on other laboratory examinations.

**Fig. 1:**
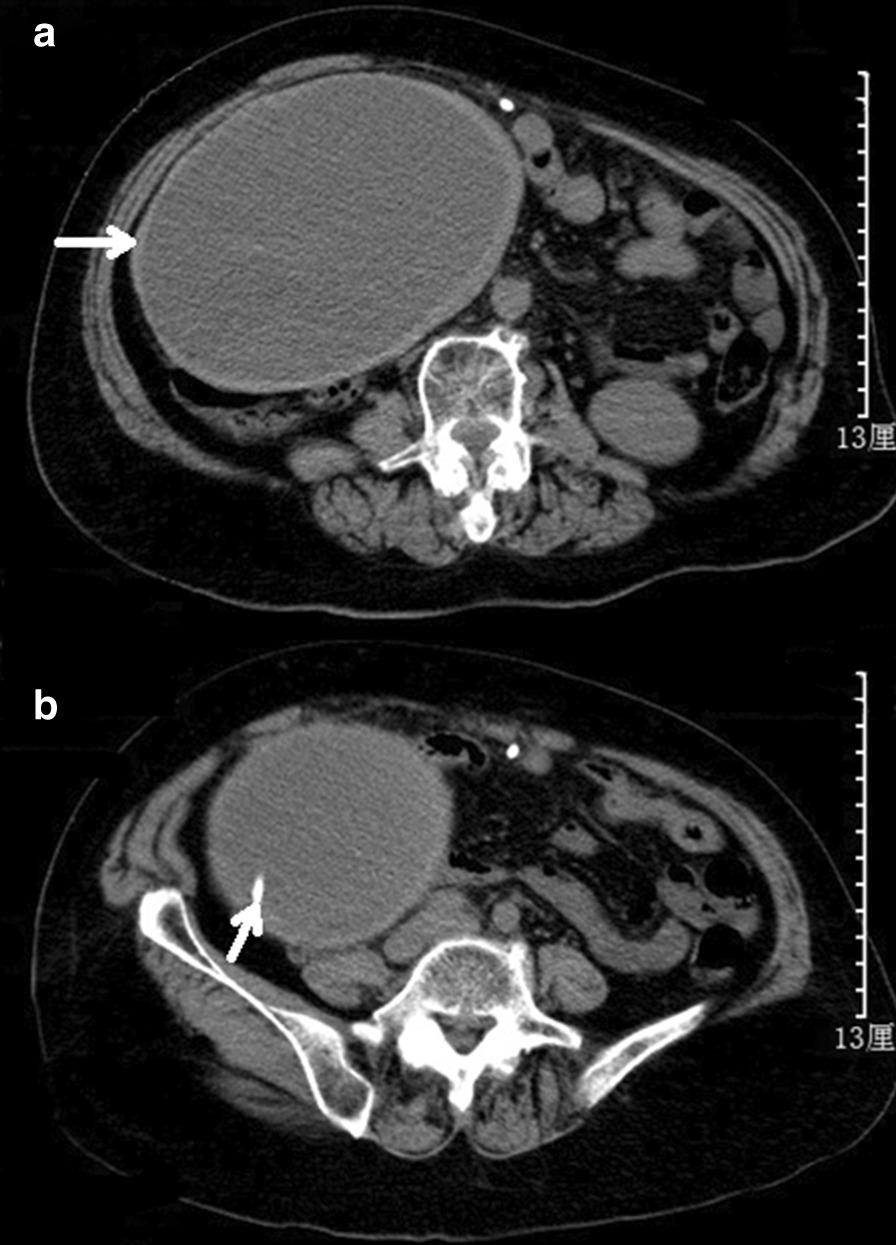
**a** Abdominal CT demonstrated a large cyst comprising a collection of homogeneous iso-density fluid in the abdominal cavity with displacement of the bowel loops to the posterior part. **b** The distal segment of the ventriculoperitoneal shunt catheter was located within the cystic mass with clear margins. The cyst and the shunt catheter were indicated by white arrows

**Fig. 2:**
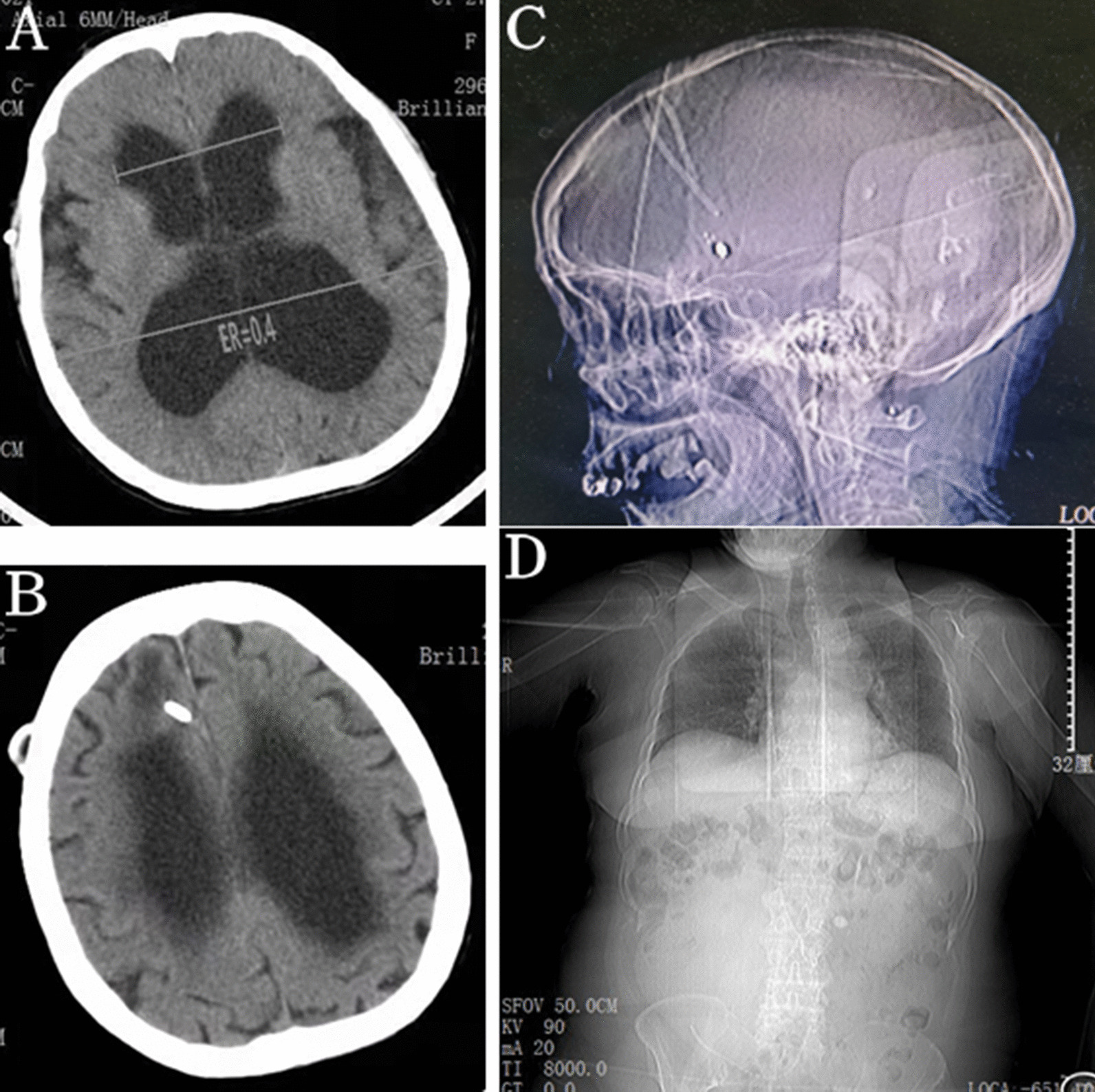
**a** Cerebral CT showed an enlarged ventricular system and its Evans Ratio was 0.4. **b** There being no interstitial cerebral edema and hemorrhage in brain or along the catheter. **c**, **d** The trajectory of the shunt on the skull and abdomen–thorax X ray did not show the catheter breakage, disconnection or migration

Unenhanced abdominal computed tomography (CT) scan indicated a large cystic collection of homogenous iso-density fluid in the right lower abdominal region with clear margins (Fig. 1a). The distal segment of the peritoneal shunt catheter was located within the cystic mass (Fig. 1b). The cystic mass, independent from the abdominal viscera, was nonloculated and 25–30 Hounsfield units in attenuation. There was no colonic obstruction. Dilation of the ventricular system was found on the cerebral CT imaging and the Evans Ratio (ER) value was 0.4 (Fig. 2a). Although the ventricular system was enlarged, interstitial cerebral edema and hemorrhage in brain or along the catheter were not found (Fig. 2b). Moreover, the trajectory of the shunt on the skull and abdomen–thorax X ray did not show the catheter breakage, disconnection or migration (Fig. 2c, d). Abdominal CSF pseudocyst was highly suspected as a preoperative diagnosis.

**Fig. 3:**
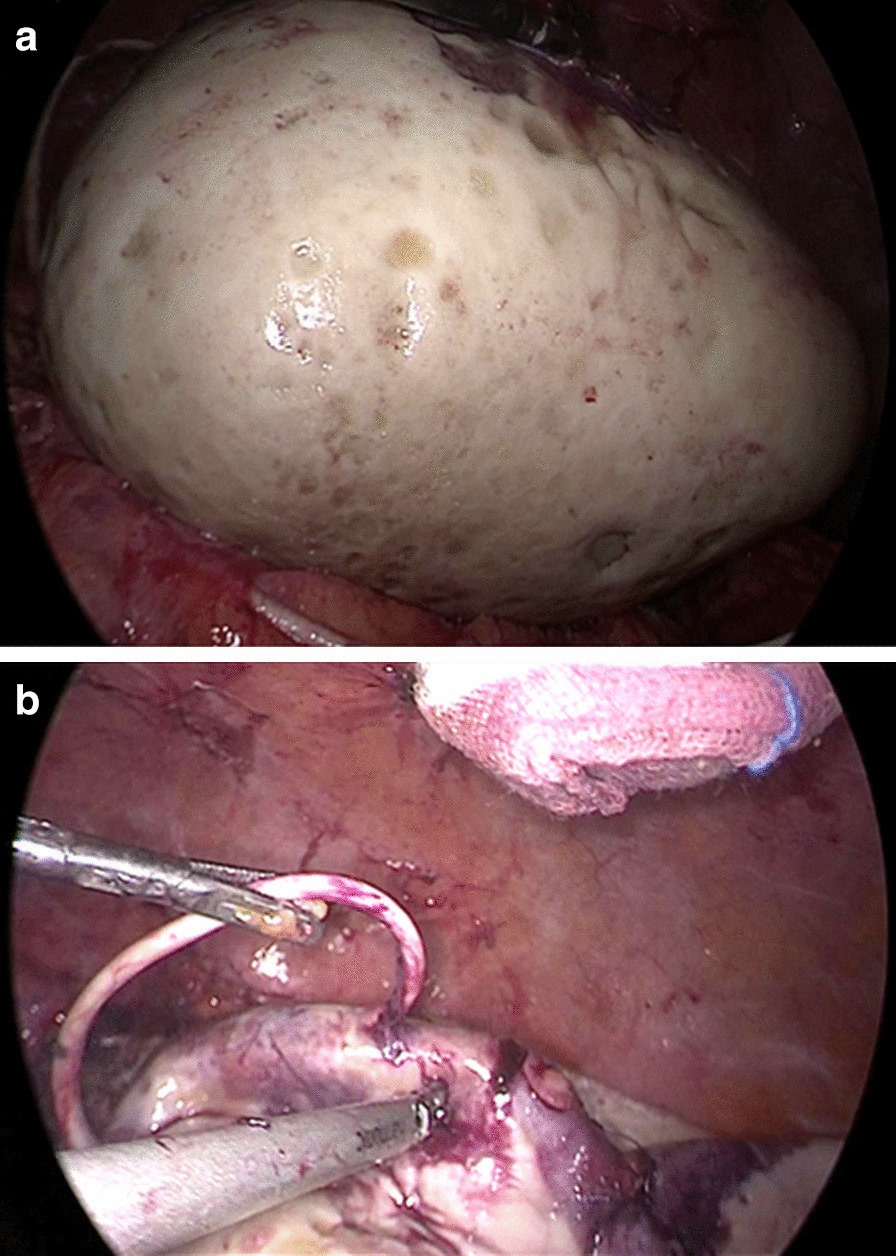
**a** The patient underwent laparoscopic cyst drainage with removal of the mass. Intraoperative finding presented a large cystic mass in the right lower abdominal cavity with clear margins. **b** The tip of the peritoneal catheter was seen within the cystic mass. The drainage fluid was chronic hemorrhage rather than clear cerebrospinal fluid. No cerebrospinal fluid dripping from the shunt catheter was found and the shunt malfunction was diagnosed

**Fig. 4:**
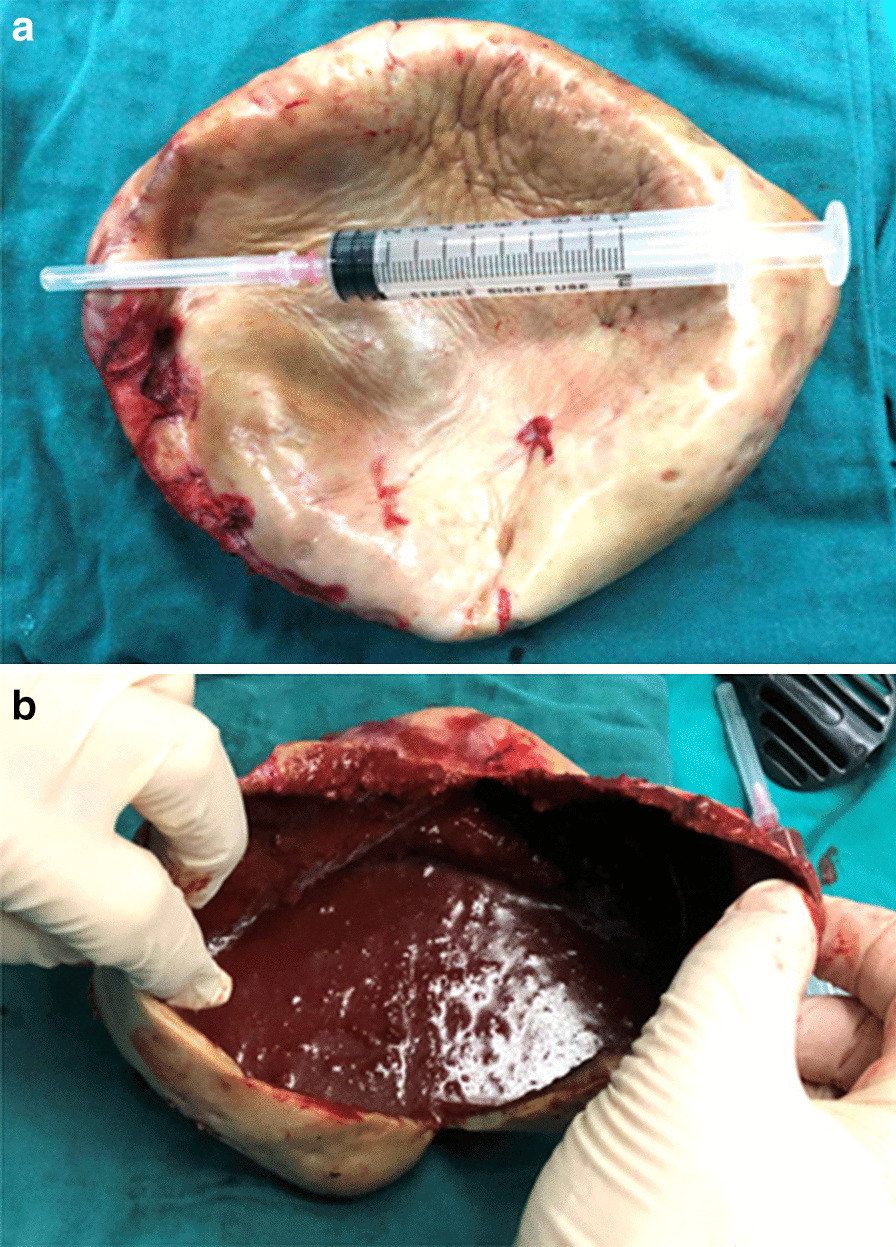
**a** The large cyst was found with thick walls and well-defined margins, without internal septa. **b** The walls were leathery and the contents in cystic mass were chronic hemorrhage

Laparoscopic cyst drainage with excision of the whole cyst was performed, 15-cm cyst which found with thick walls and organized chronic hematic content (Fig. 3). The cyst was tense and had mild adhesions. More than 2 L of the hematoma fluid was drained off. The CSF in the cyst or dripping from the end of shunt catheter was not found, and the shunt malfunction was diagnosed during operation. The distal side of peritoneal shunt catheter within the cyst was cut and then removed from her abdominal cavity. Responsible vessels associated with the cyst hemorrhage were not identified during operation and there was no definite feeding artery for the cyst. No further shunt revision was performed. A thick-walled capsule collecting hematoma fluid was further demonstrated post-surgical gross pathology specimen (Fig. 4).

Culture of the intracystic content was negative for microorganisms. Histological examination showed that the cyst wall consisted of outer fibrous tissue and inner granulation tissue without epithelial lining, and the cystic content was chronic hematoma. The patient had an uneventful postoperative course and remained asymptomatic for 8-mo follow-up.

## Discussion and conclusions

VP shunts are commonly placed to treat hydrocephalus from various causes, diverting excess CSF from the lateral ventricles into the peritoneum. The peritoneal cavity can absorb fluid rapidly and efficiently, making it an ideal site for CSF diversion [5]. Although VP shunts are usually safe and straightforward, more complications may occur along with more patients surviving and living longer after successful operation; numerous reports of various complications from the presence of the catheter in the peritoneal cavity have been published [1, 6]. Common complications include shunt obstruction, tip migration, infection, over drainage, tube disconnection, abdominal visceral perforation, omental clogging, and bowel obstruction.

Formation of abdominal CSF pseudocyst is a well recognized, but not common complication. Abdominal CSF pseudocyst, which refers to a collection of CSF in the peritoneal cavity at the distal end of the VP shunt catheter, was first noticed and understood as a complication after a VP shunt by Harsh in 1954 [3]. Although this abdominal complication is not usually life-threatening, it can cause shunt dysfunction and create diagnostic and therapeutic challenges. Therefore, knowing this particular condition is important in clinical practice. The time from the last shunting procedure to the development of this complication usually ranges from 3 weeks to 10 years [2, 4, 7]; however, there had been a reported case of abdominal CSF pseudocyst formation 21 years after VP shunt placement and our patient developed the abdominal pseudocyst 15 years after her VP shunt [8].

The most frequent symptoms and signs of abdominal CSF pseudocyst in adult patients are local abdominal signs, usually involving abdominal pain, distention and a palpable abdominal mass [9]; whereas symptoms derived from shunt malfunction and elevated intracranial pressure, such as headache, vomiting and drowsiness, are more common in pediatric patients [10]. The same is true in our case and she merely presented with abdominal complaints without neurological symptoms. No more than 30% adult patients presented with symptoms of shunt malfunction [9]. Abdominal pseudocysts following VP shunts are most often slow-growing and present with symptoms gradually [2, 4–8]. However, hemorrhagic onset in the abdominal pseudocyst can increase the pressure within the pseudocyst, and then accelerate the appearance of clinical symptoms and signs of this complication.

The imaging techniques used in the diagnosis of abdominal CSF pseudocyst are ultrosonography (US) and abdominal CT. The main criteria for diagnosis in both US and CT include the presence of an intraperitoneal fluid collection with well-defined margins and the identification of the distal tip of catheter within the collection [2–4, 11]. US has proved to be a very useful imaging technique for diagnosing and monitoring the complication because it is fast and reliable [11]; However, CT is considered more effective in the diagnosis in adult patients, especially when masses are large and deform the normal architecture of the abdomen [9]. In the present report, abdominal CT revealed a large fluid-filled collection delimited by a thick wall in the right lower abdominal region. Although measurement of attenuation values with CT characterized the contents as not pure CSF attenuation [12], the distal segment of the peritoneal shunt catheter within the cystic mass strongly suggested the preoperative diagnosis of abdominal CSF pseudocyst. The high Hounsfield units in attenuation of the cyst may be related to the increased protein content of CSF.

The management of abdominal CSF pseudocyst is still a point of discussion and no standards have been established. Many therapeutic modalities have been described as successful [4, 6, 13], for example laparotomy and wide excision of the cystic walls, paracentesis and aspiration of the cystic fluid, CT-guided or US-guided aspiration of the pseudocyst and, more recently, laparoscopic-associated lysis of the abdominal CSF pseudocyst. However, it is critically important that the presence or absence of infection must be established as it determines the definitive treatment [5, 13, 14]. Treatment strategies should be also adjusted for the patient’s overall clinical status [14–16]. Laparoscopic cyst drainage with excision of the whole cyst was performed in the present case. Surprisingly, the cyst was found with thick walls and organized chronic hematoma; the CSF in the cyst or dripping from the end of shunt catheter was not found. Because the hematoma in the cyst was chronic and degraded, the intracystic collection appeared as iso-density on abdominal CT imaging.

Surgical evacuation followed by repositioning of the catheter in the peritoneal cavity is the treatment of choice for most patients [3–6, 13–15]. Converting the shunt to being ventriculoatrial or ventriculopleural can help in some cases, if there is either pseudocyst recurrence or shunt malfunction, indicating that the peritoneal cavity is not suitable as a long-term CSF diversion location [5, 13]. However, further shunt revision was not performed for the patient due to no definite neurological signs and symptoms. It may be explained by the rebalance of CSF circulation on the condition of the slow shunt obstruction. This situation is similar to the fact that a small number of patients with idiopathic normal pressure hydrocephalus might improve without shunting [17].

While the underlying mechanisms involved in the formation of abdominal CSF pseudocyst are still unknown, inflammatory process, either sterile or infectious, is commonly regarded as the principle causative factor [5, 7, 13]. It has been further suggested that smaller or multiloculated pseudocysts tend to be infected and larger pseudocysts tend to be sterile [14, 15]. Additionally, other predisposing factors have been also described, such as prior surgical peritoneal adhesions, multiple shunt revisions, peritonitis, distal shunt migration, high CSF protein, changes in CSF absorption and allergic reactions [6, 9]. In our case, the most likely cause of her pseudocyst was a non-specific local tissue reaction against the shunt tubing material in the peritoneum in view of the absence of infectious symptoms, no evidence of infection on laboratory examinations, and lack of any inflammatory changes in surrounding tissues.

The pathological mechanism of hemorrhagic onset in the abdominal pseudocyst is not clear and no case of a hemorrhagic abdominal pseudocyst after VP shunts has been reported so far. Apart from cyst collections, the pathological features of the hemorrhagic cyst wall were identical with CSF pseudocyst wall; therefore, the hemorrhagic abdominal pseudocyst following VP shunt was most likely evolved from abdominal CSF pseudocyst. This special condition may be similar to the evolution of subdural effusion into chronic subdural hematoma (CSDH). Although bleeding in abdominal CSF pseudocyst has not been reported yet, the evolution of subdural effusion into CSDH is a well-recognized clinical phenomenon [18].

The evolution of subdural effusion into CSDH is attributed to the following reasons [18, 19]: the increase in subdural effusion that results in an increase in the subdural space, which in turn, leads to bleeding after the bridge vein is pulled; after a long period of subdural effusion, an envelope is formed gradually; bleeding after neovascularization in the membrane; changes in the properties of the effusion, small molecule inflammatory substances cause degradation of lymphocyte aggregation, leading to the formation of hematomas due to leakage of new capillaries; fibrin dissolves and increases in the effusion, leading to bleeding due to coagulation dysfunction; local hyperfibrinolysis of the outer membrane in the CSDH prevents complete hemostasis and induces rebleeding into the hematoma cavity.

Although inflammatory substances and coagulation condition of the cyst were not analyzed in this case, pathological studies on the cyst wall showed fibrous tissue with granulation tissue and neovascularization on the inner surface. The hemorrhagic event in the abdominal pseudocyst may be related to the inner granulation tissue and neovascularization. It is clear that there is more to this condition than neovascularization causing bleeding into the abdominal pseudocyst; therefore, further study and clinic observation are needed to explore this special condition.

In summary, this is the first report of hemorrhagic onset in the abdominal pseudocyst following VP shunt. This special condition can accelerate the appearance of clinical symptoms of the abdominal pseudocyst, and its mechanisms may be similar to the evolution of subdural effusion into CSDH.

## Data Availability

The dataset used during this study available from the corresponding author on reasonable request.

## Abbreviations

CSDH: Chronic subdural hematoma
CSF: Cerebrospinal fluid
CT: Computed tomography
VP: Ventriculoperitoneal
